# Strain-Induced Robust Exchange Bias Effect in Epitaxial La_0.7_Sr_0.3_MnO_3_/LaFeO_3_ Bilayers

**DOI:** 10.3390/molecules29143244

**Published:** 2024-07-09

**Authors:** Jun Zhang, Tiancong Su, Jianchun Ma

**Affiliations:** 1 Department of Chemical & Material Engineering, Lyuliang University, Lishi 033001, China; singermajianchun@126.com; 2 School of Chemistry and Materials Science of Shanxi Normal University & Key Laboratory of Magnetic Molecules and Magnetic Information Materials of Ministry of Education, Taiyuan 030006, China; tiancongs1457@163.com; 3 Institute of New Carbon-Based Materials and Zero-Carbon and Negative-Carbon Technology, Lyuliang University, Lishi 033001, China

**Keywords:** complex oxide system, La_0.7_Sr_0.3_MnO_3_/LaFeO_3_, epitaxial strain, exchange bias

## Abstract

The ground state of correlated electrons in complex oxide films can be controlled by applying epitaxial strain, offering the potential to produce unexpected phenomena applicable to modern spintronic devices. In this study, we demonstrate that substrate-induced strain strongly affects the coupling mode of interfacial magnetic moments in a ferromagnetic (FM)/antiferromagnetic (AFM) system. In an epitaxial bilayer comprising AFM LaFeO_3_ (LFO) and FM La_0.7_Sr_0.3_MnO_3_ (LSMO), samples grown on a LaAlO_3_ (LAO) substrate exhibit a larger exchange bias field than those grown on a SrTiO_3_ substrate. Our results indicate a transition in the alignment of magnetic moments from perpendicular to collinear due to the large compressive strain exerted by the LAO substrate. Collinear magnetic moments at the LSMO/LFO interface generate strong exchange coupling, leading to a considerable exchange bias effect. Thus, our findings provide a method for tailoring and manipulating the orientations of magnetic moments at the FM/AFM heterogeneous interface using strain engineering, thereby augmenting methods for exchange bias generation.

## 1. Introduction

The exchange bias (EB) effect, discovered by Meiklejohn and Bean, has drawn much attention from the scientific community due to its crucial role in spin valve structures [1], which are essential in numerous spintronic devices, such as magnetic sensors, magnetoresistive read heads, and magnetic random access memories [2,3,4,5,6,7,8]. EB is typically produced by an interfacial interaction between ferromagnetic (FM) and uncompensated antiferromagnetic (AFM) materials [9]. When a sample is cooled below the AFM Néel temperature (T_N_) under a static magnetic field, the magnetic hysteresis (M–H) loop shifts away from the center of symmetry at a zero magnetic field and generates an EB field, H_EB_ [10]. H_EB_ is given by (H_R_ + H_L_)/2, where H_R_ and H_L_ are points on the right- and left-field axes, respectively, where the M–H loop intersects.

With technological advancements, various approaches have been proposed for generating and manipulating EB. These approaches include generating the strain-engineering-induced EB effect through self-assembly in a single (La,Sr)MnO_3_ thin film [11]; the ionic liquid-gated manipulation of O^2−^ and H_EB_ in a cobalt oxide/manganese oxide bilayer [12]; the induction of EB via interfacial charge transfer and orbital reconstruction in paramagnetic LaNiO_3_-based (LaMnO_3_/LaNiO_3_) superlattices [13]; the full electric control of EB in Si/SiO_2_/Pt/Co/NiO/Pt devices [14]; the spin–orbit torque switching of FM magnetization and modulation of EB in Pt/Co/IrMn trilayers [15]; the pressure-engineering-based tuning of H_EB_ and blocking temperature in two-dimensional FePSe_3_/Fe_3_GeTe_2_ van der Waals heterostructures [16]; photocontrol of EB in Co_90_Fe_10_/BiFeO_3_ heterostructures [17]; and other methods for controlling EB [18,19,20,21]. Although the aforementioned studies reveal rich characteristics and fascinating physical phenomena, they primarily focus on controlling magnetic moments in the FM or paramagnetic phase while neglecting the effect of manipulating magnetic moments in the AFM phase for generating EB.

LaFeO_3_ (LFO) exhibits a canted G-type AFM phase with a T_N_ of up to approximately 740 K. Due to this extremely high T_N_, LFO is a promising material for applications in EB-related devices. Seo et al. found a notable approximately ten-fold increase in H_EB_ when LFO was partially diluted with Ni atoms in the (001)-oriented Co/LFO/La(Ni_0.1_, Fe_0.9_)O_3_ thin film. In this system, the structural domain boundary became corrugated due to nonmagnetic defects, indicating the strong influence of the domain boundary configuration on EB [2]. Hallsteinsen et al. observed an induced magnetic moment of 1.6 ± 0.40 µB/Fe for nominally AFM LFO extending 2–4 interface layers in a (111)-oriented La_0.7_Sr_0.3_MnO_3_ (LSMO)/LFO/SrTiO_3_ (STO) bilayer [22]. Subsequently, in the same bilayer system, they observed that the LFO AFM spin axis gradually shifts from the out-of-plane to the in-plane direction with increasing LFO layer thickness [23]. Bruno et al. found that net magnetic moments of Fe were induced in the first few unit planes of LFO near the interface with LSMO in a (001)-oriented LSMO/LFO/STO bilayer, with these Fe magnetic moments coupled in an antiparallel manner to Mn magnetic moments [24]. Gopalarao et al. successfully fabricated c axis-oriented LSMO/LFO bilayers on an LAO substrate with varying LSMO layer thickness, which ranges from 30 to 200 nm. The value of the out-of-plane lattice constant of LSMO was found to decrease with its increasing thickness. Magnetization measurements and analysis show that the lattice strain and the AFM LFO layer strongly affected the magnetic properties of the LSMO layer, especially at low thickness [4]. The coercive field (H_c_) of all aforementioned LSMO/LFO bilayers was higher than that of a single layer of LSMO; however, no EB effect was observed.

In this study, a complex oxide system of LSMO is selected as the FM layer while LFO is selected as the AFM layer, to construct a LSMO/LFO bilayer on the (001)-oriented STO (denoted as the LSMO/LFO/STO bilayer) and (001)-oriented LaAlO_3_ (LAO; denoted as the LSMO/LFO/LAO bilayer) crystal substrates via reflection high-energy electron diffraction (RHEED)–assisted pulsed laser deposition (PLD). The LSMO/LFO/STO bilayer exhibits a negligible EB effect when subjected to a small compressive strain from the STO substrate. In contrast, considerable H_EB_ is induced in the LSMO/LFO/LAO bilayer under a large compressive strain from the LAO substrate. Experimental results indicate that the alignment of magnetic moments at the FM/AFM interface gradually changes from perpendicular to collinear with increasing epitaxial strain. This collinear coupling generates a strong pinning field at the bilayer interface, resulting in the formation of a prominent EB effect. The proposed method for controlling EB in FM/AFM heterostructures via strain engineering has broad application prospects in the field of spintronic devices.

## 2. Results and Discussion

**Figure 1:**
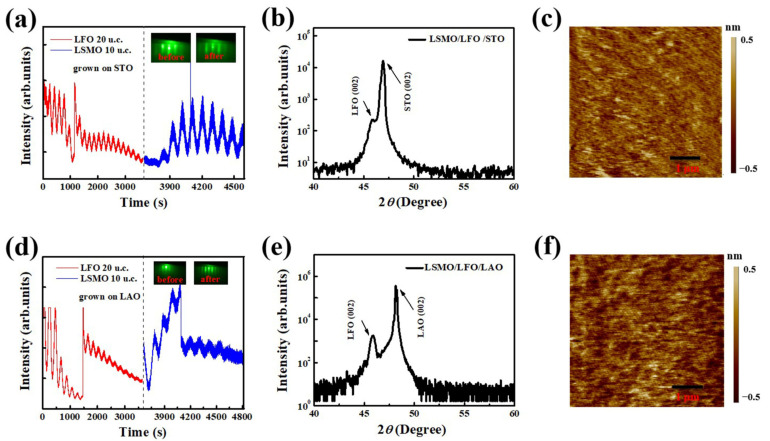
(**a**,**d**) Integral RHEED oscillation curves, dashed lines represent the dividing line between the LSMO and LFO oscillation curve; (**b**,**e**) XRD θ–2θ scans in the range of 40–60°; and (**c**,**f**) surface morphologies over a 5 µm × 5 µm scan range for LSMO/LFO/STO and LSMO/LFO/LAO bilayers, respectively. Insets of (**a**,**d**) display RHEED patterns recorded before and after bilayer preparation.

The RHEED oscillation curves and diffraction patterns obtained during the preparation of the LSMO/LFO/STO and LSMO/LFO/LAO bilayers, are presented in Figure 1a,d, respectively. Before deposition, the typical RHEED patterns of the two substrates reveal strong Laue diffraction spots, suggesting that the bare substrates exhibit smooth surface flatness. After the deposition of LSMO/LFO bilayers, the clean diffraction spots gradually disappear and transform into straight parallel streaks, indicating that high-crystalline-quality bilayers with a layer-by-layer growth mode are formed during the deposition process. This observation is further supported by the cyclical oscillation curves. The LFO bottom layers (LSMO top layers) exhibit 20 (10) noticeable oscillations, revealing the configuration of perfect LSMO 10 u.c./LFO 20 u.c. bilayers.

Figure 1b,e present X-ray diffraction (XRD) θ–2θ scans measured around the (002) diffraction peaks of the substrates for the LSMO/LFO/STO and LSMO/LFO/LAO bilayers, respectively. The crystalline orientations exhibit full alignment along the (001) direction, and both the (002) diffraction peaks of LFO can be observed on the left side of the substrate (002) peaks. The lattice constant is 3.78 Å (3.905 Å) for the LAO (STO) substrate and 3.926 Å for the bulk LFO. Therefore, the lattice mismatch of LFO deposited on the LAO and STO substrates, calculated using the formula $\frac{a_{\mathrm{sub}.}-a_{\mathrm{LFO}}}{a_{\mathrm{sub}.}}\times 100\%$, is −3.86% and −0.53%, respectively. The larger compressive strain imposed by the LAO substrate increases the value of the c axis lattice constant and shifts the (002) peak toward a smaller θ–2θ direction. Because the LSMO layer has a thickness of only 10 u.c. and its lattice constant is similar to those of the LAO and STO substrates, no peaks corresponding to this phase can be clearly observed in the XRD spectra.

Figure 1c,f display the surface morphologies of bilayers. The average root-mean-square roughness of the LSMO/LFO/STO and LSMO/LFO/LAO bilayers is 1.1 and 1.08 Å, respectively, indicating an atomically smooth surface. Therefore, all measurement results, namely, the RHEED oscillation curves, θ–2θ scans, and surface roughness measurement results, support the high crystalline quality and strict monitoring of the growth process in the construction of LSMO/LFO bilayers, providing a reliable foundation for the study of interfacial EB.

**Figure 2:**
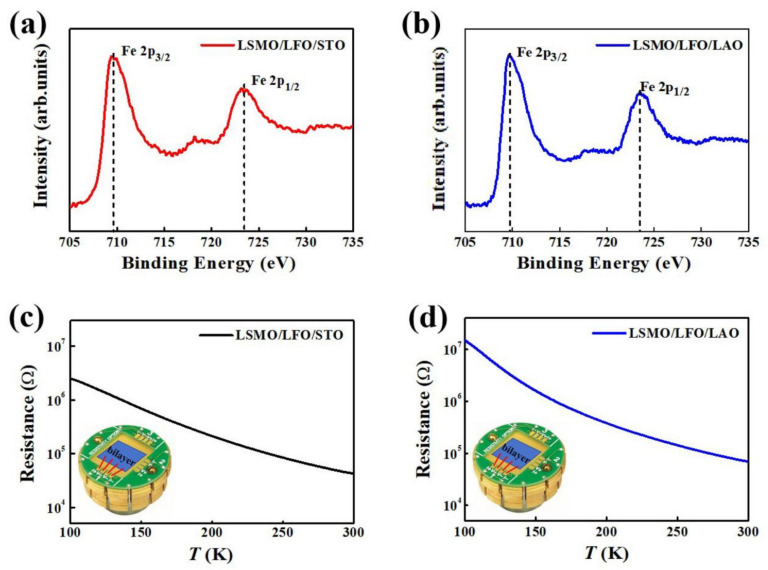
(**a**,**b**) Fe 2p XPS spectra and (**c**,**d**) corresponding R–T curves of LSMO/LFO/STO and LSMO/LFO/LAO bilayers, respectively. Insets of (**c**,**d**) depict circuit structure diagrams for measuring in-plane resistance using a standard four-lead method.

To further elucidate the valence states of Fe in different strained bilayers, Fe 2p X-ray photoelectron spectroscopy (XPS) analysis was performed, and the results are presented in Figure 2a,b. For the LSMO/LFO/STO bilayer, two characteristic peaks are observed at binding energies of approximately 709.65 (Fe 2p_3/2_) and 723.81 eV (Fe 2p_1/2_), with a shake-up satellite peak around 718.4 eV. For the LSMO/LFO/LAO bilayer, the binding energies of Fe 2p_3/2_ and Fe 2p_1/2_ estimated in the present study are approximately 709.75 and 723.52 eV, respectively. Furthermore, a satellite peak is observed at around 718.5 eV, assigned to the Fe^3+^ state in oxide forms for both the samples [25,26,27]. As determined from the above experimental results, the binding energies of Fe 2p_3/2_, and Fe 2p_1/2_, and the corresponding shake-up satellite peak for the LSMO/LFO bilayer grown on the LAO substrate are very close to those of the bilayer grown on the STO substrate, suggesting that different substrate-induced strains have a negligible effect on the valence state of Fe in the LFO AFM layer. 

Figure 2c,d illustrate the resistance–temperature (R–T) curves of the LSMO/LFO/STO and LSMO/LFO/LAO bilayers, respectively. The resistance of both these bilayers decreases as the temperature increases, and the corresponding R–T curves indicate insulating behavior when the thickness of the LSMO top layer is less than 10 u.c., which is consistent with a previous study [28]. In addition, the M–H loops of the LFO 20 u.c. single layer grown on the STO and LAO substrates were measured at 5 K (Figure S1). The results demonstrate that both the M–H loops exhibit similar weak ferromagnetism, indicating an uncompensated magnetic moment distribution in the two AFM single layers.

**Figure 3:**
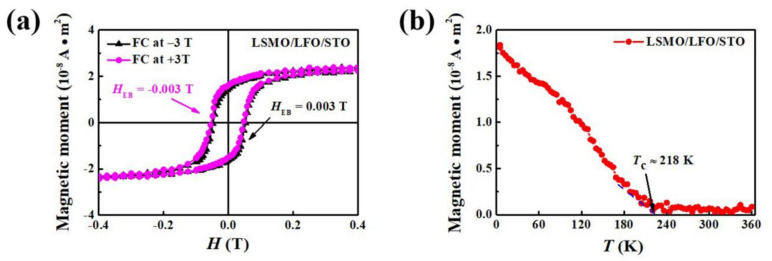
M–H loops and M–T curve for LSMO/LFO/STO bilayer. (**a**) M–H loops at 5 K after treatment via +3 and −3 T FC processes; (**b**) M–T curve measured from 5 to 360 K with an applied magnetic field of +0.3 T. Sample surface is parallel to the applied magnetic field in M–T curve measurements.

Figure 3a displays the M–H loops of the LSMO/LFO/STO bilayer measured using a field-cooling (FC) treatment. The FC M–H loop measurements were performed after cooling samples under the +3 T and −3 T cooling field from room temperature (RT) to 5 K at a rate of 5 °C/min; additionally, the cooling field was applied along the in-plane (100) direction of the substrate (parallel to the sample edge). Under a +3 T cooling field, the center of the M–H loop shifts along the magnetic field axis toward negative fields, whereas under a −3 T cooling field, the center of the M–H loop shifts along the magnetic field axis toward positive fields. Although the bilayer generates the EB effect under the action of the cooling field, H_EB_ is very small (only approximately 0.003 T), which is negligible compared with its H_c_ of approximately 0.051 T. In a previous study, Folven et al. investigated interfacial coupling in a LFO/LSMO/STO bilayer. For a bilayer with a lateral dimension larger than 1 μm, the coupling of magnetic moments at the FM/AFM interface primarily favors the perpendicular alignment (spin-flop coupling). Thus, spin-flop coupling at the bilayer interface generates weak exchange coupling and thus produces a negligible EB effect [29]. Figure 3b presents the temperature-dependent magnetization (M–T) curve of the LSMO/LFO/STO bilayer. To measure the M–T curve with an FC protocol, samples were cooled from 360 to 5 K under an applied magnetic field of +0.3 T. Based on a previous study [11], the FM Curie temperature (T_c_) of the bilayer is estimated to be approximately 218 K, determined using the intersection of the slope of the M–T curve with the abscissa.

**Figure 4:**
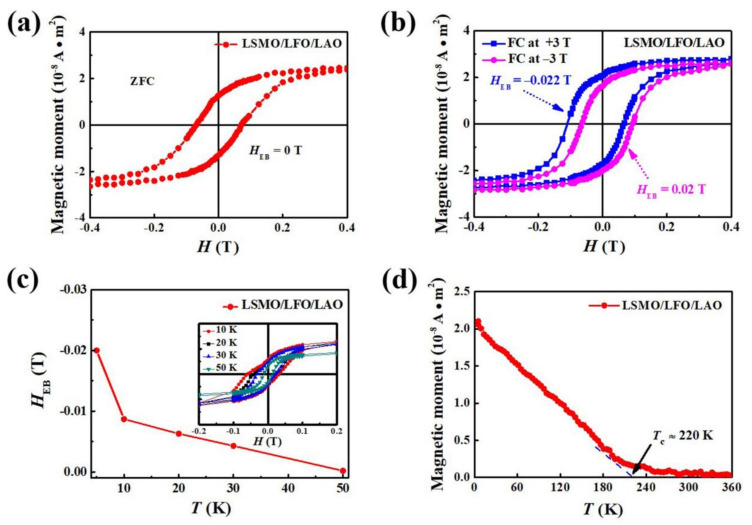
M–H loops, T–H_EB_ plot, and M–T curve for LSMO/LFO/LAO bilayer. (**a**) M–H loops measured at 5 K after treatment via ZFC process; (**b**) M–H loops measured at 5 K after treatment via +3 T and −3 T FC processes; (**c**) temperature dependence of H_EB_ under +3 T applied field (inset displays the temperature dependence of the M–H loop); and (**d**) M–T curve measured from 5 to 360 K under an applied magnetic field of +0.3 T.

Applying epitaxial strain to complex oxide thin films is a powerful tool for controlling the ground state of correlated electron systems. In particular, it is widely believed that the magnetic properties of complex oxides are strongly correlated with a substrate-induced strain. Based on this theory, we prepared an LSMO/LFO bilayer on a LAO substrate, which provides a larger compressive strain in the thin film. We then obtained its M–H loops to further investigate the relationship between EB and epitaxial strain. Figure 4a displays the zero-field-cooling (ZFC) M–H loop of the LSMO/LFO/LAO bilayer measured at 5 K. The M–H loop does not shift from the center of the coordinate axis, and the corresponding H_EB_ is equal to zero, suggesting that there is no EB effect under the ZFC protocol. Subsequently, M–H loops at 5 K of the bilayer after ±3 T FC treatment were measured, and the results are presented in Figure 4b. Note that the H_EB_ of the LSMO/LFO/LAO bilayer reaches approximately 0.02 T, which is almost an order of magnitude larger than that of the LSMO/LFO/STO bilayer. In addition, the H_c_ of the LSMO/LFO/LAO bilayer reaches 0.085 T, which is higher than that of the LSMO/LFO/STO bilayer (0.051 T). This indicates that the large compressive strain imposed by the LAO substrate may cause the interfacial magnetic moments of our sample to change their alignment from perpendicular to collinear, thereby producing a strong pinning field at the FM/AFM interface and eventually increasing H_EB_ and H_c_. This phenomenon is also supported by the different magnetization reversal processes discussed later. 

The temperature dependence of H_EB_ in the bilayer under a +3 T cooling field is displayed in Figure 4c, revealing a blocking temperature of approximately 50 K. To further confirm the magnetism of the FM layer in our bilayer, the M–T curve is presented in Figure 4d. It can be seen that its FM T_c_ of approximately 220 K is similar to that of the LSMO/LFO/LAO bilayer (218 K), indicating that the strain imposed by different substrates has a minimal impact on the T_c_ of the LSMO FM layer.

**Figure 5:**
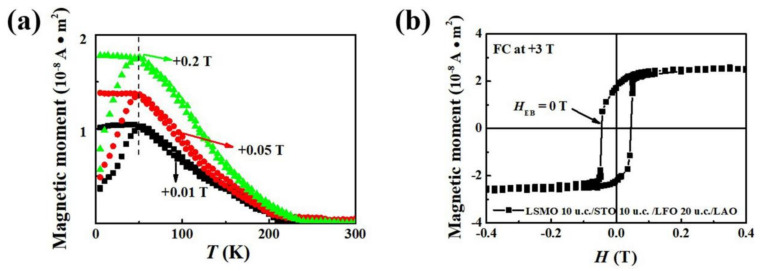
(**a**) M–T curves of the LSMO/LFO/LAO bilayer measured under different magnetic fields (+0.01, +0.05, and +0.2 T) after ZFC and FC processes and (**b**) M–H loop of LSMO 10 u.c./STO 10 u.c./LFO 20 u.c./LAO thin film measured at 5 K after treatment with +3 T FC processes.

To eliminate the spin glass state in our experiment, the M–T curves were measured at various field strengths (0.01, 0.05, and 0.2 T) in LSMO/LFO/LAO bilayer after the FC and ZFC procedures, as shown in Figure 5a. The ZFC curves exhibit a peak (T_P_) and a bifurcation between the ZFC and FC M–T curves below the irreversibility temperature (T_irr_). Furthermore, both temperatures greatly decrease with increasing magnetic field strength for the spin glass system, suggesting that the frozen state is clearly suppressed by a strong field. However, in our experiment, these two characteristic temperatures remain nearly constant as the measurement field increases, suggesting no clear evidence of the spin glass behavior [9]. 

The magnetic properties of the LSMO layer are highly sensitive to substrate-induced strain, as reported by Cui et al. [30]. This implies that EB may appear if substrate-induced strain propagates into the LSMO layer through the underneath LFO layer. To investigate this, we inserted a 10 u.c. STO nonmagnetic layer between the LSMO and LFO layers (namely LSMO 10 u.c./STO 10 u.c./LFO 20 u.c./LAO thin film) and measured the M–H loop, as shown in Figure 5b. The exchange coupling between LSMO and LFO disappears due to the isolation of the STO nonmagnetic layer, rendering the H_EB_ value close to zero. This observation is consistent with the results obtained for the LSMO 10 u.c./STO 10 u.c./LFO 10 u.c./LAO thin film. This conclusion indicates that the EB effect in our bilayer originates primarily from magnetic coupling interactions between the LSMO and LFO interface rather than from strain-induced effects in the LSMO ferromagnetic layer.

**Figure 6:**
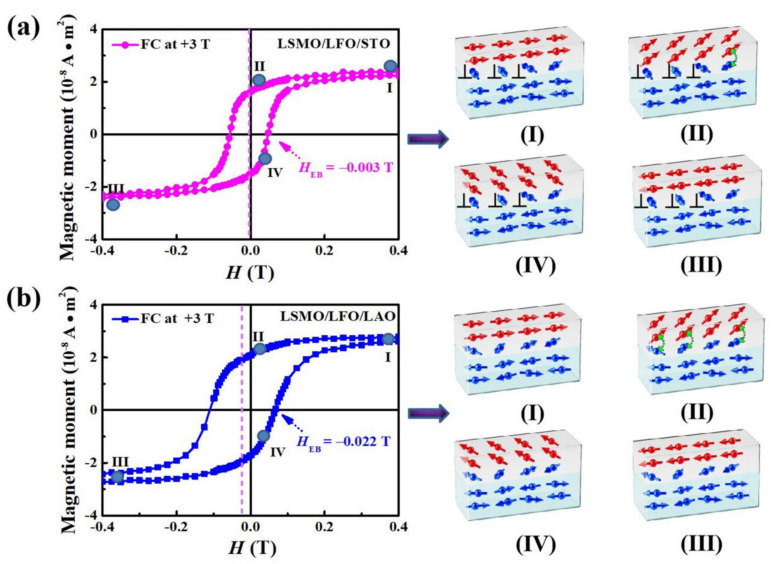
(**a**,**b**) Different magnetization reversal processes (steps I–IV) in the M–H loops of Figure 3a and Figure 4b, respectively. Dark green dots in M–H loops represent different magnetization states, while green double arrows indicate the strong AFM coupling of magnetic moments at the FM/AFM bilayer interface and red (blue) arrows represent FM (AFM) magnetic moments. ⊥ denotes that magnetic moments are perpendicular to each other. Dashed lines represent the position where the M–H loops deviates from the origin of the coordinates.

Next, we investigate potential changes in interfacial magnetic moments from the initial to the final magnetization states using M–H loops with +3 T FC treatment (examples are shown in Figure 3a and Figure 4b). The magnetization reversal processes are depicted schematically in Figure 6a (denoted as the LSMO/LFO/STO bilayer) and Figure 6b (denoted as the LSMO/LFO/LAO bilayer). Blue arrows in both figures, not completely antiparallel, indicate an uncompensated magnetic moment distribution in AFM layers. All FM magnetic moments in both the bilayers rotate in the direction of the positive field up to saturation when an external magnetic field is applied. However, the magnetic moments of the FM/AFM interface in the LSMO/LFO/STO bilayer exhibit the perpendicular or near-perpendicular alignment if the bilayer is subjected to a small compressive strain (Figure 6a, step I). When the magnetic moments of the samples are reversed by applying an external magnetic field, there is no coupling between the perpendicularly aligned magnetic moments (indicated by ⊥), and the angle between the near-perpendicular magnetic moments of Fe and Mn is larger than 90°, which produces stable AFM exchange coupling (indicated by green double arrows) at the interface and prevents the magnetic moments of the LSMO layer from reversing continuously along the direction of the external magnetic field (Figure 6a, steps II–III) [24]. When the magnetic moments of the bilayer are reversed (Figure 6a, steps III–IV), the angle between the near-perpendicular magnetic moments of Fe and Mn is smaller than 90°, which produces weak FM exchange coupling at the interface. This causes the magnetic moments in the LSMO layer of the LSMO/LFO/STO bilayer to switch easily, ultimately leading to a negligible EB effect. 

For the LSMO/LFO/LAO bilayer, the perpendicular alignment of magnetic moments at the FM/AFM interface is destroyed under a large compressive strain (Figure 6b, step I). When an external magnetic field is applied to reverse the magnetic moments of the bilayer, as illustrated in step II of Figure 6b, a pinning force due to strong interfacial AFM coupling (indicated by green double arrows) prevents them from continuously reversing along the direction of the external magnetic field [24]. As they attempt to return from a negative saturation state to a positive saturation state (Figure 6b, steps III–IV), the FM layer switches easily because most interfacial magnetic moments at this time exhibit weak FM exchange coupling. This particular magnetization reversal process may cause the M–H loop to shift towards the negative field direction, leading to a noticeable EB effect. Previously, EB has been effectively controlled through various methods, such as the substrate strain-induced EB effect in YSMO/LSMO heterostructures [30], ionic liquid-gated switching of EB in SrFeO_3−x_/La_0.7_Sr_0.3_MnO_3_ bilayers [31], interfacial charge transfer–induced EB in LaMnO_3_/LaNiO_3_ superlattices [13], and magnetoelastically induced perpendicular EB in CoO/CoPt multilayer films [32]. In these manipulation processes, EB arises from the asymmetric pinning action of uncompensated AFM magnetic moments at the FM–AFM interface on FM magnetic moments. In contrast, EB disappears when compensated AFM magnetic moments systematically pin FM magnetic moments at the FM–AFM interface. This mechanism contrasts with our model, where magnetic moments at the FM–AFM interface change their alignment from perpendicular to collinear owing to a large compressive strain, eventually leading to a robust EB effect.

## 3. Materials and Methods

High-quality epitaxial LSMO/LFO bilayers (LSMO as the top layer and LFO as the bottom layer) were deposited on the (001)-oriented STO and LAO crystal substrates via PLD with a 248 nm KrF excimer laser. The STO (a_STO_ = 0.390 nm) and LAO (a_LAO_ = 0.379 nm) substrates (5 mm × 5 mm × 0.5 mm) were used to induce compressive strain. Stoichiometric LSMO and LFO targets, each with a diameter of 2 in, were purchased from Hefei Kejing Materials Technology Co., Ltd. (Hefei, China). Prior to growth, all substrates were heated to 725 °C for 30 min under a high background vacuum of 1 × 10^−5^ Pa. During deposition, the LFO bottom layer and LSMO top layer were fabricated under identical conditions. The optimal preparation parameters of the target–substrate distance and laser energy density were 5.5 cm and 4.5 J/cm^2^, respectively. The laser pulse frequency was set at 2 Hz, the deposition temperature was maintained at 725 °C, and an oxidizing atmosphere with an oxygen partial pressure of 5.3 Pa was maintained during PLD by attaching an automatic air intake system. Subsequently, the samples underwent annealing for 60 min in a high-oxygen environment (an oxygen partial pressure of 4 × 10^5^ Pa) before being cooled to RT at a rate of 15 °C/min to minimize their oxygen vacancies. Growth qualities were monitored in situ via RHEED, enabling the precise control of sample thickness at the unit cell (u.c.) level and the accurate characterization of the growth dynamics. The crystalline orientation of the obtained bilayers was investigated using XRD (Rigaku Ultima IV-185 diffractometer (Akishima, Japan), θ–2θ geometry using Cu–Kα radiation), and the surface roughness of the samples was quantified using atomic force microscopy (Bruker, Dimension Icon, Billerica, MA, USA) with the tapping mode. The valence states of Fe ions were assessed using XPS (Kalpha, Thermo K-Alpha, Waltham, MA, USA) with an Al Kα monochromatic source (1486.6 eV) operated at 12 kV and 3 mA under a vacuum pressure of 1.0 × 10^−7^ Pa. High-resolution spectra were acquired with a pass energy of 20 eV (energy step of 0.04 eV) at RT using a hybrid mode with a spot size of 700 μm × 300 μm. All samples exhibited sufficient conductivity without any charging effects. The binding energies of all spectra were calibrated to a value of 284.8 eV for the C1s core level. The spectra were collected and processed using the Thermo Scientific Avantage XPS software (Version 5.96). The electrical transport characteristics were investigated using a physical property measurement system (Quantum Design Dynacool-9) by employing a standard four-lead measurement method and applying a temperature change rate of 5 °C/min. The magnetic properties were analyzed using a superconducting quantum interference device (MPMS-XL-5), with an in-plane applied magnetic field. To evaluate the magnetization of the prepared samples, virgin substrate data were measured, and the linear contribution from diamagnetic STO and LAO was subtracted from the experimental data.

## 4. Conclusions

Herein, we investigated the influence of the strain imposed by the substrate on the EB effect in the epitaxial LSMO/LFO/STO and LSMO/LFO/LAO bilayers. For the bilayers subjected to a small compressive strain, interfacial coupling primarily favors the perpendicular alignment of magnetic moments in the AFM and FM layers, leading to weak exchange coupling and negligible H_EB_. In contrast, for bilayers subjected to a large compressive strain, a partial collinear alignment of AFM and FM moments is observed at the bilayer interface, resulting in the robust exchange coupling and noticeable EB effect. The use of epitaxial strain to control the magnetic moment alignment at the FM/AFM bilayer interface provides a method to control the exchange coupling strength and effectively modulate the EB effect.

## Data Availability

The data used to support the findings of this study are available from the corresponding authors upon request.

## Supplementary Materials

The following supporting information can be downloaded at: https://www.mdpi.com/article/10.3390/molecules29143244/s1, Figure S1: M–H loops of (a) LFO 20 u.c./STO single layer and (b) LFO 20 u.c./LAO single layer. All M–H loops were measured at 5 K.
