# Supplementary material for: Strain-Induced Robust Exchange Bias Effect in Epitaxial La0.7Sr0.3MnO3/LaFeO3 Bilayers
Source: Molecules. 2024 Jul 9;29(14):3244. doi: 10.3390/molecules29143244 (PMC11279207; doi:10.3390/molecules29143244)
Supplement: Supplementary file 1 [file molecules-29-03244-s001.zip › molecules-3056717-supplementary.pdf]

Supporting information

Strain-Induced Robust Exchange Bias Effect in Epitaxial $La_{0.7}Sr_{0.3}MnO_3/LaFeO_3$ Bilayers

Jun Zhang [1,*,†], Tiancong Su [2,3,†] and Jianchun Ma [1]

1 Department of Chemical & Material Engineering, Lyuliang University, Lishi 033001, China; singermajianchun@126.com
2 School of Chemistry and Materials Science of Shanxi Normal University & Key Laboratory of Magnetic Molecules and Magnetic Information Materials of Ministry of Education, Taiyuan 030006, China; tiancongs1457@163.com
3 Institute of New Carbon-Based Materials and Zero-Carbon and Negative-Carbon Technology, Lyuliang University, Lishi 033001, China
* Correspondence: zhangjunclz@126.com
† These authors contributed equally to this work.

Figures S1(a) and S1(b) show the *M–H* loops of the LFO 20 u.c. single layer grown on STO and LAO substrates, respectively. The results demonstrate that both *M–H* loops exhibit similar weak ferromagnetism, indicating an uncompensated magnetic moment distribution in the two antiferromagnetic single layers.

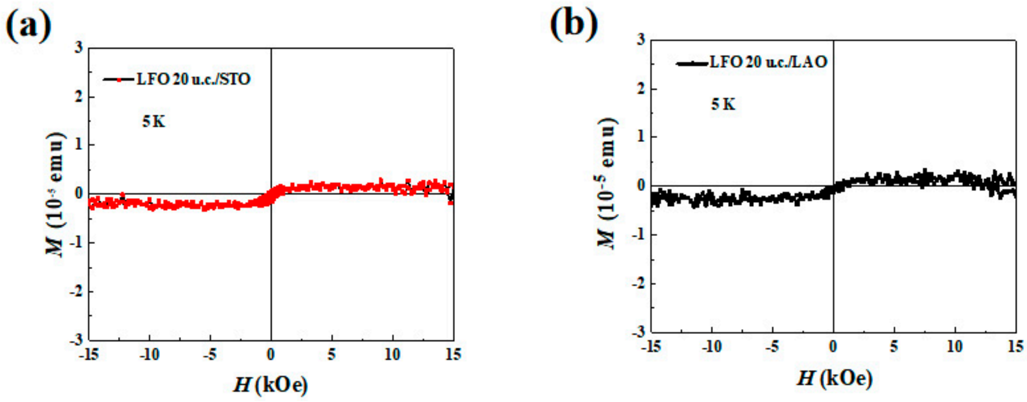

Figure S1. *M–H* loops of (a) LFO 20 u.c./STO single layer and (b) LFO 20 u.c./LAO single layer. All *M–H* loops were measured at 5 K.
